# Photosystem II Assembly from Scratch

**DOI:** 10.3389/fpls.2015.01234

**Published:** 2016-01-12

**Authors:** Thilo Rühle, Dario Leister

**Affiliations:** ^1^Plant Molecular Biology, Department of Biology, Ludwig-Maximilians-University MunichMunich, Germany; ^2^Department of Plant and Environmental Sciences, Copenhagen Plant Science Centre, University of CopenhagenCopenhagen, Denmark

**Keywords:** PSII assembly, PSII, PSII complex, assembly factor, synthetic bacterium, forward genetic screen, reverse genetic, chloroplast

*Construction of a functional Photosystem II (PSII) in cyanobacteria and chloroplasts depends on the action of auxiliary factors, which transiently interact with PSII intermediates during assembly. In addition to a common PSII structure and a conserved set of PSII assembly factors, cyanobacteria, and higher plants have evolved additional, clade-specific assembly factors. Most such factors in cyanobacteria and chloroplasts have been identified by “top-down” approaches (forward and reverse genetics), which involved genetic disruption of individual components in the assembly process and subsequent characterization of the ensuing phenotypic effects on the respective mutant lines/strains. In contrast, a “bottom-up” strategy, based on the engineering of a synthetic bacterium with a plant-type PSII, has the potential to identify all assembly factors sufficient to make a functional plant PSII*.

Photosystem II (PSII) is a water-plastoquinone photo-oxidoreductase, which is found in cyanobacteria and their endosymbiotic descendants, the chloroplasts. Light-driven water splitting and subsequent electron transfer steps are carried out with the assistance of non-proteinaceous cofactors. Thus, the PSII monomer harbors a Mn_4_CaO_5_ cluster, chloride, bicarbonate, 1-2 hemes, 1 nonheme iron, 35 chlorophyll *a* molecules, 2 pheophytins, 11 β-carotenes, and 2 plastoquinones (Umena et al., 2011), all of which are embedded in a shell made up of at least 20 proteins (Shen, 2015) that determine their correct positioning and relative orientation. Several PSII-associated lipids have been identified in crystal structures and might also be important for functionality (Mizusawa and Wada, 2012; Kansy et al., 2014). The structural core of PSII is conserved between chloroplasts and cyanobacteria (Allen et al., 2011). However, the oxygen-evolving complex in cyanobacteria contains subunits U and V, which are replaced by Q, R, P, and Tn in higher plants (Bricker et al., 2012). Furthermore, in contrast to the soluble, peripherally attached phycobilisomes found in cyanobacteria, green photosynthetic eukaryotes have evolved integrated light-harvesting complexes and lack phycobilisomes (Hohmann-Marriott and Blankenship, 2011; Figure 1A).

**Figure 1:**
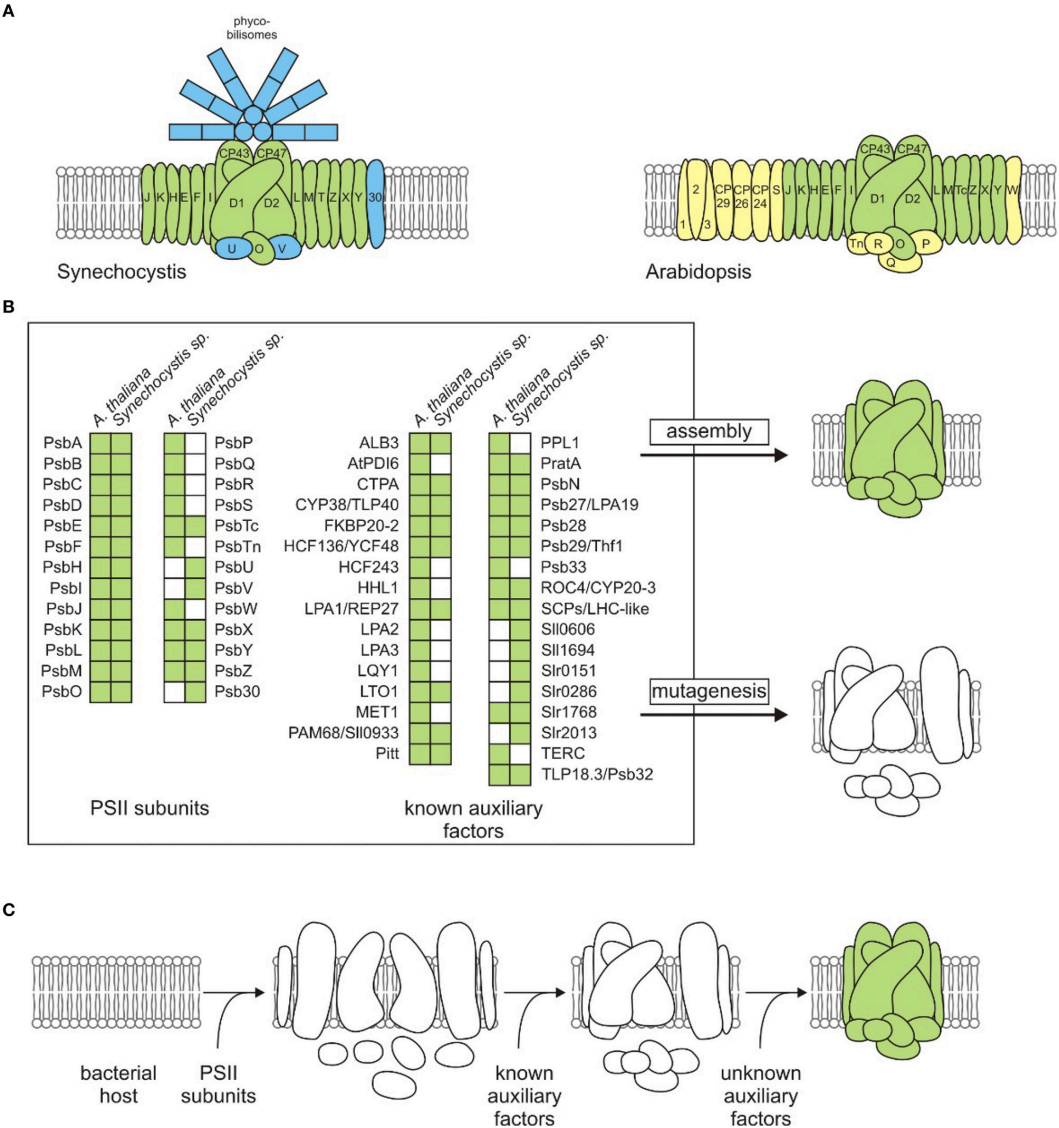
**Strategies for identifying auxiliary factors in PSII assembly. (A)** Subunit compositions of cyanobacterial (*Synechocystis*) and plant thylakoid (*Arabidopsis*) PSII complexes. Components shown in blue or yellow are only found in *Synechocystis* or *Arabidopsis*, respectively. Light-harvesting proteins Lhcb1, 2 and 3 are labeled with numbers (1, 2, and 3). **(B)** PSII subunits and known PSII auxiliary factors. The list of auxiliary factors has been adapted from Nickelsen and Rengstl (2013) and Järvi et al. (2015), and the recently identified auxiliary factors MET1 (Bhuiyan et al., 2015) and Slr0151 (Yang et al., 2014) have been added. Note that for simplicity, no distinction has been made between factors necessary for assembly, repair or supercomplex formation, and Deg/FtsH proteases are not listed. The classical strategy for study of the assembly process is based on mutagenesis of single components in an intact system. Subsequent analyses focus on the identification of a specific loss of function in a photosynthetic organism. Green and white squares indicate presence and absence of a component listed for each organism, respectively. **(C)** Generation of a synthetic, bacterial host strain carrying a functional plant-type PSII. The design of the synthetic approach largely depends on the choice of the host organism. In the case of a cyanobacterial host, endogenous PSII subunits or auxiliary factors must be deleted or replaced by the plant-type subunits/factors, whereas the presence of essential cofactors (see text), in addition to subunits and auxiliary factors, must be assured for the assembly of a functional, plant-type PSII in a non-photosynthetic bacterium. White and green PSII cartoons in **(B,C)** indicate un- or partially assembled and fully assembled PSII complexes, respectively.

In accordance with its structural complexity, the assembly of PSII is an elaborate and highly coordinated process, which depends on the action of a network of assembly factors and the fabrication of distinct metastable modules during the course of assembly (see for reviews on this topic: Nixon et al., 2010; Nickelsen and Rengstl, 2013; and papers in this special issue of Frontiers in Plant Science). Several modules common to cyanobacterial and chloroplast PSII assembly processes have been described and are characterized by transient binding of specific assembly factors. An additional level of complexity arises from the fact that PSII is susceptible to photodamage, with subunit D1 being the primary target (Kato and Sakamoto, 2009), as replacement of damaged D1 entails partial disassembly of PSII and reassembly of a functional complex. This repair mechanism features some distinct intermediates, but otherwise involves steps and assembly factors that are shared with the *de novo* assembly pathway (Järvi et al., 2015).

It has become increasingly clear in recent years that the set of assembly factors is largely conserved between cyanobacteria and chloroplasts (Nickelsen and Rengstl, 2013). However, higher plants have extended their inventory during evolution, giving rise to new, plant-specific factors, such as the D1 stabilization factor HCF243 (Zhang et al., 2011) or the repair factors PPL1 and LQY1 (Ishihara et al., 2007; Lu et al., 2011; Figure 1B). Furthermore, the consequences of disruption of conserved auxiliary factors sometimes differ between the two systems. For instance, *Arabidopsis* PAM68 and its cyanobacterial counterpart (Armbruster et al., 2010), as well as HCF136 (Meurer et al., 1998) and the cyanobacterial homolog YCF48 (Komenda et al., 2008), participate in the early steps of PSII assembly, but their absence has more severe effects on photosynthesis in plants. Thus, to understand the evolutionary diversification of assembly factors, it is crucial to study chloroplast and cyanobacterial PSII assembly in parallel.

## Classical or “top-down” methods of identifying PSII assembly factors

Extensive efforts have been made over the past several decades to identify auxiliary factors involved in PSII assembly, and a large number have been found by screening *Synechocystis* sp. PCC 6803 (hereafter *Synechocystis*), *Chlamydomonas reinhardtii*, or *Arabidopsis thaliana* (hereafter *Arabidopsis*) mutant collections for PSII-defective mutants. This type of genetic approach has emerged as a powerful strategy, since single components of an initially intact system can be functionally deleted without directly altering other elements in the assembly process. Subsequent studies then allow the in-depth characterisation of the effects of the loss of a specific function in the assembly process.

### Forward genetic screens

A classical way to screen for mutants defective in PSII is based on the observation of a high-chlorophyll fluorescence (HCF) phenotype in mutagenized *Arabidopsis* plants, which can be recognized when leaves are exposed to UV light in the dark (Meurer et al., 1996). Several assembly factors, including HCF136 and HCF243 (stabilization of D1 and assembly of the reaction center), LPA1 (D1 integration), LPA2 and LPA3 (integration of CP43) were identified in this manner (Meurer et al., 1998; Peng et al., 2006; Ma et al., 2007; Cai et al., 2010; Zhang et al., 2011). The sensitivity of this screening method for PSII mutants can be further increased by measuring photosynthetic parameters of large mutant collections under varying growth conditions using automatic pulse-amplitude modulation (PAM) of chlorophyll fluorescence (Varotto et al., 2000) or imaging PAM technologies (Ajjawi et al., 2010). This type of approach has led to the identification of several novel factors such as LPA19, PAM68, LQY1, HHL1, and PSB33 (Armbruster et al., 2010; Wei et al., 2010; Lu et al., 2011; Jin et al., 2014; Fristedt et al., 2015). However, forward screens are both labor-intensive and time-consuming, and more than 90% of nuclear genes in *Arabidopsis* have at least one homolog (Armisén et al., 2008). In many cases, duplicated genes code for proteins with redundant functions, and for that reason are inaccessible to classical forward screening techniques.

### Reverse genetic screens

The increasing accumulation of information on entire genomes, transcriptomes, and proteomes in public databases, together with the availability of indexed libraries of *Arabidopsis* mutant lines with mutations in almost every gene, and the establishment of efficient methods for gene silencing, and genetic engineering by means of homologous recombination in cyanobacteria, have made it possible to apply reverse genetic strategies to both cyanobacterial and eukaryotic photosynthetic model organisms and selectively knock out whole gene families. In one such case, the *Synechocystis* genome database was searched for TPR (tetratricopeptide repeat) genes (Klinkert et al., 2004) and the ORFs were systematically mutagenized. Of the 22 putative TPR proteins identified, PratA and Pitt were shown to be important for Mn^2+^ transport to D1 (Stengel et al., 2012) and for early steps in photosynthetic pigment-protein complex formation (Schottkowski et al., 2009), respectively. Interestingly, the green lineage-specific TPR-domain-containing protein MET1 was recently shown to assist in PSII supercomplex formation and in PSII repair (Bhuiyan et al., 2015). A further example of the power of reverse genetics is the screen of the lumenal immunophilin family in *Arabidopsis*, which comprises at least 16 FKBP (FK-506 binding proteins) and cyclophilins that are known to function as protein chaperones or foldases. Two of these, CYP38/TLP40 and FKBP20-2, were found to be involved in early PSII biogenesis and PSII-LHCII assembly, respectively (Lima et al., 2006; Fu et al., 2007; Sirpiö et al., 2008).

### Potential and limitations of top-down approaches

It is obvious that additional PSII assembly auxiliary factors will be identified in forward as well as reverse genetic screens. But the latter are becoming more important owing to major advances in rates of data generation and data mining brought by “omics” technologies. Additionally, methods for large-scale quantification of transient protein interactions of PSII subunits or known PSII auxiliary factors with unknown proteins (Braun et al., 2013) will provide a more thorough understanding of the components and their function in the assembly process.

However, an inherent limitation of top-down approaches is that they allow only those factors that are *required* for a given process to be identified. To define the suite of assembly factors and additional auxiliary factors *sufficient* for PSII assembly, an entirely different strategy is needed, in which all structural PSII proteins and auxiliary factors are brought together and the final outcome of their interplay (PSII assembly) can be monitored. This “bottom-up” concept cannot be optimally implemented in commonly used model organisms like *Arabidopsis*, maize, barley or tobacco, as “brute force” genetic approaches (like shot-gun complementation) are not feasible in these organisms and non-photosynthetic propagation of mutants is difficult to achieve, if at all. Consequently, new tactics have to be adopted to speed up and complement research on PSII assembly. Such efforts must be directed at the discovery of the complete set of auxiliary factors sufficient to make a functional plant-type photosystem and are outlined below.

## A novel “bottom-up” approach: Generation of a synthetic bacterium with a functional plant-type PSII

In this context, microbial cell systems possess unsurpassed advantages over plants in several respects. They are fast growing (with doubling times as short as 20 min), they have small genomes that are easy to manipulate, and do not show genetic compartmentalization. The ideal host system for study of the assembly of a plant-type PSII via a “bottom-up” approach should fulfill the following requirements: (i) be able to synthesize such essential non-proteinaceous cofactors as Chl *a*, pheophytin, heme and β-carotene, (ii) possess an appropriate electron acceptor for PSII, and (iii) a lipid composition similar to that found in thylakoids, (iv) be capable of heterotrophic growth, and (v) intermediates should accumulate in mutants that are dysfunctional in the assembly process. Notably several cyanobacteria meet virtually all these criteria, which is a reflection of their evolutionary relationship to chloroplasts. However, in the case of a cyanobacterial host like *Synechocystis* endogenous PSII subunits and known, conserved auxiliary factors must be deleted, because they could interfere with the plant-type PSII subunits or auxiliary factors to be tested. This would entail the use of either a sequential, marker-less deletion strategy (Viola et al., 2014 and references within) or a time-saving, multiplex editing strategy recently described for bacterial strains, but not yet established in polyploid cyanobacteria (Ramey et al., 2015). Alternatively, cyanobacterial PSII genes can be directly replaced by their (appropriately optimized) plant counterparts in order to keep untranslated regulatory regions and operon structures intact (Viola et al., 2014). A plant-type PSII can then be constructed stepwise by mimicking the chloroplast assembly process. Known and normally short-lived assembled modules, for instance the D2-Cyt *b*_559_ complex, the reaction-center complex lacking both CP47 and CP43 or the reaction-center complex lacking CP43 alone (Müller and Eichacker, 1999; Komenda et al., 2004; Dobáková et al., 2007; Nixon et al., 2010), can serve as checkpoints after introducing known plant-type assembly factors into a cyanobacterial strain expressing the respective plant-type subunits found in each module. As the ultimate goal, unknown factors required for the building of each PSII module can be identified by complementation assays with cDNA libraries derived from higher plants (Figure 1C). An initial benchmark for this kind of approach would be the successful assembly of the minimal PSII core of the five subunits D1, D2, α, and β subunits of Cyt *b*_559_ and PsbI, which is able to perform charge separation (Nanba and Satoh, 1987).

### Critical aspects

The bottom-up strategy is inherently appealing, but there are significant uncertainties. While top-down strategies rely on the disruption of a single component of a complex system followed by the characterisation of the effects attributable to loss of that component, the success of the bottom-up strategy critically depends on the ability to monitor the completion of each step in the assembly process. This is the vital prerequisite for the identification of novel assembly factors introduced by shot-gun complementation approaches by perhaps only incremental advances in the assembly process. Nevertheless, in light of the tremendously fast progress in sequencing, gene-synthesis and gene-editing technologies, such synthetic biology-related approaches will sooner or later enrich the field of thylakoid assembly research.

## Funding

This work was funded by a grant from the Ludwig-Maximilians-University (LMUexcellent) and by the German Science Foundation (DFG, grant LE 1265/20-1).

### Conflict of interest statement

The authors declare that the research was conducted in the absence of any commercial or financial relationships that could be construed as a potential conflict of interest.
